# Exploring the antenatal care challenges faced during the COVID-19 pandemic in rural areas of Indonesia: a qualitative study

**DOI:** 10.1186/s12884-023-05495-8

**Published:** 2023-03-16

**Authors:** Mekar Dwi Anggraeni, Rahmi Setiyani, Endang Triyanto, Asep Iskandar, Desiyani Nani, Amin Fatoni

**Affiliations:** 1 Nursing Department, Faculty of Health Sciences, Universitas Jenderal Soedirman, Purwokerto, Indonesia; 2 Chemistry Department, Faculty of Sciences, Universitas Jenderal Soedirman, Purwokerto, Indonesia

**Keywords:** Antenatal care, COVID-19, Indonesia, Pregnancy, Rural areas

## Abstract

**Introduction:**

The COVID-19 pandemic affected almost all healthcare services in Indonesia, including antenatal care (ANC). Pregnant women were a vulnerable group during the pandemic since the Indonesian government’s policies at the time influenced the delivery of ANC services, particularly in rural areas. Investigating the ANC challenges faced during the pandemic from the perspectives of pregnant women and healthcare providers is important for our understanding of ANC provision. This study, therefore explores barriers to ANC appointments faced during the COVID-19 pandemic in rural areas of Indonesia from the perspectives of pregnant women and health care providers.

**Methods:**

This was a qualitative exploratory descriptive study involving 31 participants, consisting of 25 pregnant women and six healthcare providers who were selected via a purposive sampling method. Thadeus and Maine’s Three Delays Model was used as the theoretical framework. Data were collected between March and August 2021, through two focus group discussions (FGDs), ten in-depth interviews, and field notes. Data were analyzed using a thematic analysis method.

**Results:**

Three themes describing barriers to ANC during the COVID-19 pandemic in rural areas of Indonesia emerged from this study. Those themes were: (1) The fear of being infected with COVID-19, related to anxiety, perceived vulnerability, and the desire to protect oneself and loved ones; (2) The stay-at-home policy, related to transport barriers and restricted social activity; and (3) Re-designed ANC services, related to ANC adjustments, high-risk pregnancies, insufficient information, and adherence to COVID-19 preventive behaviors.

**Conclusion:**

Based on the Three Delays Model, several challenges to carrying out ANC during the COVID-19 pandemic in rural areas of Indonesia were identified. These findings demonstrate the need to formulate and implement ANC packages to facilitate pregnant women’s access to health care services.

## Introduction

In 2020, the COVID-19 pandemic was declared an international public health problem [1]. The virus spread massively to all regions of Southeast Asia, with the total number of confirmed cases and deaths in Indonesia up to September 3rd 2021 reaching 4,116,890 and 134,930 respectively [2]. These figures meant that Indonesia ranked 13 globally in terms of total COVID-19 confirmed cases [3]. The pandemic affected all aspects of life in Indonesia, including routine health services such as antenatal care (ANC). Pregnant women were one of the most vulnerable groups during the pandemic [4], and, despite the disruption, they were still encouraged to engage in ANC during the period because of the massive health benefits associated with ANC. Despite this, ANC coverage in Indonesia has decreased over the past two years. Antenatal care coverage among pregnant Indonesian women was 88%, 88.5%, and 84.6% in 2018, 2019, and 2020 respectively [5–7]. The noticeable decline in ANC coverage in 2020 might be due to the pandemic. The Indonesian government reported the first case in Indonesia on March 2nd, 2019. Following that, the Indonesian Obstetrics and Gynecologists Association announced that 20% of MMR cases in the country between the beginning of 2020 and August 2021 were related to COVID-19 [8]. A similar study in Brazil found that 13.19% of maternal deaths in 2020 were linked to COVID-19 [9].

ANC is essential care that should be provided to all pregnant women. The main aim of ANC is to ensure that every pregnancy ends in the delivery of a healthy infant without any undesirable effects on the health of women, and this is achieved through health promotion, disease prevention, early detection, and management of complications and existing diseases [10]. There is less ANC coverage for pregnant women living in developing countries than those in developed countries, particularly in rural areas that lack healthcare providers and facilities [11] Irregular ANC is associated with complications during pregnancy, delivery, and puerperium for both mother and infant, including preeclampsia, eclampsia, anemia, preterm birth, low birth weight, and stillbirth [12]. Occasionally, pregnancy triggers diseases such as pregnancy-induced hypertension, gestational diabetes, pre-eclampsia, and eclampsia [13].

Although most maternal deaths linked to COVID-19 have occurred in pregnant women with comorbidities, previous studies have confirmed that COVID-19 in pregnant women may cause severe outcomes such as abortion [14] and increased maternal morbidity and mortality, pre-eclampsia, and preterm birth [15]. One study carried out in India found that during the pandemic, the number of deliveries taking place in institutional hospitals reduced by 45.1%, while high-risk pregnancies increased by 7.2%, admission to intensive care units saw a 2.5-fold increase, and one-third of pregnant women experienced inadequate ANC. The authors stated that the principal reasons for delayed health-seeking behaviors were lockdown and fear of transmission, and this resulted in 44.7% of pregnancies developing complications during that period [16]. Another study found that access to and continuity of ANC, particularly with regard to the early detection of COVID-19 symptoms, is highly recommended [17]. This could be because pregnant women tend to be relatively immunodeficient which could worsen an infection and lead to bad outcomes for mothers and fetuses [18]. It is clear to see that ANC is hugely important among pregnant women.

Attending ANC appointments was more challenging during the COVID-19 pandemic worldwide. One study carried out in India found that of 144 pregnant women, two thirds reported at least one barrier to ANC during the pandemic, with one third reporting fear, and more than three quarters refused ultrasound assessments and blood tests [19]. Another study identified the main barriers to ANC during the pandemic as being health facility-related barriers, poor quality of care, government regulations, anxiety, and fear of infection [20]. Another study added closing hospital/healthcare centers, the involvement of private health sectors, lack of transport, and fear of transmission from health care providers to be the major barriers to ANC during the pandemic [21]. The authors of one study cited lack of social support from husband, restricted face-to face interaction with health professionals, lack of information on COVID-19, and high uncertainty about pregnancy and birth during the pandemic as being the main ANC barriers [22]. Psychosocial challenges such as social isolation, lack of information about neonatal care, lack of public transport, and lack of up-to-date information related to COVID-19, particularly among women in low-and middle income countries were cited as the main factors in another study [23]. Pregnant women from low socio-economic classes with pre-existing pregnancy complications were more likely to experience barriers to ANC [19]. To date, little is known about the ANC challenges faced by Indonesian women during the pandemic, particularly those in rural areas who already faced a lot of barriers to ANC. Since ANC challenges during the COVID-19 pandemic in rural areas of Indonesia have not yet been sufficiently explored from the perspective of pregnant women and their healthcare providers, the knowledge provided in this study could be used to develop an appropriate and safe ANC program for pregnant women living in rural areas.

## Methods

### Aim

The aim of this study was to explore the ANC challenges during the COVID-19 pandemic in rural areas of Indonesia.

### Study design

A qualitative exploratory descriptive design was applied since it was deemed the best way of answering the who, what, and where of experiences and of obtaining insights from participants about an inadequately understood phenomenon [24, 25]. The qualitative method emphasizes deep understanding, complexity, and details of the phenomena under study, and the researcher was actively involved in the research process. The Three Delays Model developed by Thaddeus and Maine was used as the framework for this study [26].

### Setting

Indonesia is the most populous nation in Southeast Asia. It also has one of the highest MMR rates in the region [27], with around 305 MMR incidents occurring per 100,000 live births [28]. Indonesia is divided into 38 provinces and is home to over 300 ethnic groups. The biggest ethnicity in Indonesia is Javanese which accounts for around 41.71% of the total population, the majority of whom live in rural areas [29]. The third highest number of MMR cases in the country are found in Central Java Province. Data were collected in Banyumas District, the area with the sixth highest rate of MMR cases in the province. There are 17 hospitals and 30 primary health centers (PHCs) in Banyumas District, however, ANC coverage there remains lower than the national average. The district is one of the lowest 10 districts for ANC coverage in Central Java Province [30]. The study was conducted between April and August 2021. Most pregnant women attend ANC in a primary health center according to Ministry of Health policies, but high-risk pregnancies may be referred to secondary or tertiary hospitals, depending on the severity of their pregnancy-related problems.

### Participants

Included in this study were pregnant women and healthcare providers living or working in rural areas. A purposive sampling technique was used to select the study participants. The inclusion criteria for pregnant women were those in their second or third trimester who had attended ANC at least three times in a PHC. Participants were recruited through the PHC list of pregnant women and all those meeting the inclusion criteria who agreed to participate in the study were invited for data collection. Women whose pregnancy was deemed high-risk were excluded from the study. The inclusion criteria for healthcare providers were those who worked in a PHC providing ANC that had had at least three years’ experience. Healthcare providers were selected purposively and sent a formal invitation to participate in the study by post. A total of 31 participants including 25 pregnant women and six healthcare providers agreed to participate in the study and were invited to attend focus group discussions (FGDs) and in-depth interviews.

### Data collection

Data collection only began after the researchers received approval from the Institutional Review Board (IRB) of the Faculty of Medicine, Universitas Jenderal Soedirman. Data were collected by the researchers, all of whom are faculty members of the nursing department in the Faculty of Health Sciences, Universitas Jenderal Soedirman. Most researchers were of Javanese ethnicity and familiar with the principles of qualitative research and interview guidelines. Prospective participants were selected with consideration given to their social, economic, and demographic characteristic, including age, level of education, employment status, and parity for pregnant women, and length of work experience for healthcare providers, since a greater diversity of study participants allows for a more in-depth exploration and analysis of a phenomena. To adhere the Declaration of Helsinki for studies involving human subjects and ethical issues, each participant was informed of the study’s purpose, procedure, and risks, and told that participation was voluntary and that they could quit at any time without facing any consequences. Pregnant women choosing to withdraw from the study knew that they would continue to receive ANC in their PHCs as usual. The researchers also guaranteed confidentiality and anonymity of participants’ information.

Two FGDs were conducted for the purpose of this study. Twelve pregnant women participated in first FGD and nine pregnant women took part in the second. The study was conducted between March and August 2021. Because the COVID-19 situation worsened in Indonesia in July 2021, it was not possible to conduct another FGD and so the data collection method switched to in-depth interviews conducted through online interviews. At this point, the researchers conducted ten in-depth interviews to four pregnant women and six healthcare providers using video calls. The researchers initially met each participant in the PHC. There they introduced themselves, informed the participants of the study’s aims, and shared contact information. The researchers then contacted each proposed participant via WhatsApp to gain their permission to participate in the study. Each participant was provided with an electronic informed consent form before starting the interviews, and any participant struggling to fill out the electronic version was provided with a hard copy. Researchers verbally told all participants that the interviews would be recorded and transcribed.

Information related to demographic characteristics was collected before the FGDs began. The FGDs were conducted in the Bahasa Indonesia language using interview guidelines by a researcher who was an expert in community health nursing and familiar with the FGD process. The researcher leading the FGDs holds a doctoral degree in community health and has experience in carrying out qualitative research. The first FGD was conducted in the meeting room at the PHC and the second FGD was conducted in the meeting room of the Sub-District Government Office. The in-depth interviews were conducted by a researcher holding a magister degree in community health and has experience in carrying out qualitative research. The researchers introduced themselves as faculty members in Universitas Jenderal Soedirman and let participants know they were free to talk with them.

All discussions and interviews were audio recorded for verbatim transcription. During the FGDs, researchers encouraged participants to share their knowledge, feelings, and experiences regarding ANC coverage during the COVID-19 pandemic, and another researcher observed and took notes to supplement the audio recorded FGDs. The observations were used to confirm the results from the FGDs and in-depth interviews to enrich the data. Only researchers and participants attended the FGD sessions to ensure confidentiality and privacy.

In a qualitative study, data saturation is reached when extending the interviews delivers no new data and all founded codes are recurrent [24]. In this study, saturation was achieved with the 31st participant. At this point, the researchers ended the data collection, meaning there were 31 participants were involved in this study, ten of whom met on an individual basis and the others of whom attended the two focus groups. Each interview and FGD lasted around 60 and 90 min respectively. No repeat interviews were carried out and two prospective participants declined to participate, citing busy schedules.

### Research instrument

Data were collected using a semi-structured interview guideline that was developed based on the literature review on ANC in the COVID-19 pandemic situation. A panel of experts consisting of a maternity nurse and two lecturers from the maternity department reviewed the interview guidelines for their relevance to the study’s purpose. The interview guidelines were also verified and pilot tested on five pregnant women not involved as study participants. The FGDs and in-depth interviews used semi-structured questions, and probes were used to elicit further descriptions of challenges and experiences.

### Data analysis

Data were analyzed using a thematic analysis method included six phase framework for doing a thematic analysis included familiarized themselves with the data, produced initial codes, searched for themes, evaluated the themes, defined and named the themes, and made the study report [31]. Three researchers analyzed the data independently. These researchers analyzed the data manually. First, the data were transcribed the day after collection and were analyzed by the researchers to be familiar with the data. Data collection and data analysis were conducted simultaneously. The transcribed verbatim was carefully read and re-read by the researchers, and the researchers provided the participants with the opportunity to check the verbatim. Second, all the transcribed verbatim were carefully and precisely examined line by line to find initial codes, and then were categorized based on the meaning similarity. Data was cleaned by eliminating all distinguishable information, and the researchers created initial codes before interviewing the next participant. Third, the researchers developed sub-themes and themes based on the categorized data. They categorized the codes with associated meanings into one group called sub-themes and considered their significance. They then categorized all codes and categories into central categories called themes. Fourth, researchers worked together to evaluate the draft of themes in biweekly meetings. The entire research team then discussed the coding, categories, sub-themes, and themes and organized them into charts. Fifth, each member of the research team agreed upon the names of the themes and sub-themes, and some participants were asked to read the study results and give feedback about whether the themes accurately portrayed their views. Finally, the research team discussed and agreed upon the names of the final themes that emerged from this study before making the final report. The last phase is write-up study report following the consolidated criteria for reporting qualitative research (COREQ) [32]. The demographic characteristics of the participants were presented in frequency, percentage, median, minimum, and maximum.

### Trustworthiness

To assess the quality of the study data, the researchers used Lincoln and Guba’s trustworthiness criteria [24]. Most researchers in this study were Javanese and native residents of the region under investigation, and this helped gain participants’ confidence and familiarity. The study included pregnant women and healthcare providers varying in age, parity, level of education, working status, and working experience to help identify different views and concepts and heighten credibility. During the study’s execution, a qualitative research specialist observed the data collection and processing, and two qualitative researchers analyzed the data independently. The data collection, data analysis, and theory generation process can be audited, which heightens the study’s dependability. The researchers attempted to avoid subjective prejudices by recording all FGDs and interviews, keeping accurate field notes, and avoiding interfering with the data analysis results, leading to better confirmability within the study. The results were shared with participants who were asked to confirm whether they accurately reflected their experiences, leading to better transferability within the study.

### Rigor

The interview guidelines were based on a literature review focusing on ANC coverage during the COVID-19 pandemic. The draft of interview guidelines was checked for clearness and modified based on suggestions made by a panel of experts and pregnant women independent of the study. It was then tested on five pregnant women and two healthcare providers. The data were translated into English at each stage of the data analysis process. Participants were given the opportunity to carry out member checking to validate the data and were asked for their opinions regarding the themes. The entire research team read and discussed the data to confirm the right information was reported. Three researchers involved in this study had previous experience in conducting and publishing qualitative studies, which further helped to ensure the rigor of the qualitative data.

### Ethical consideration

Ethical approval for this study was granted by the IRB of the Medical Faculty, Universitas Jenderal Soedirman No 1204/KEPK/III/2017. Permissions were also granted by district assemblies and the District Ministry of Health. Researchers explained the research purposes, procedures, risks, participants’ expected role, and the voluntary nature of participation in the study to each participant. Participants were also informed that they were free to leave the study any time and that this would not affect any healthcare services received by them. Either a written or electronic informed consent form was obtained by each participant before involvement in the study and agreement to audio record the discussion or in-depth conversation was also obtained. All participants’ data were documented with codes to guarantee anonymity and only the research team had access to the research data to ensure confidentiality.

## Results

The majority of pregnant women included in the study were between 20 and 35 years old (52%), multiparous (68%), housewives (68%), and had graduated from senior high school (40%). The healthcare providers included in the study consisted of three midwives (50%), a nurse (16.7%), a head of the PHC (16.7%), and a doctor (16.7%). The mean age of the healthcare providers was 41.83 years, with a range of 34–52 years. The majority of healthcare providers had more than 11 years’ work experience (66.7%). Half of healthcare providers had completed either a bachelor’s degree or diploma of midwifery (Table 1). The details of participant’s demographic data were provided in Table 2.

**Table 1:** The participant’s demographic data

Variables	Frequency (%)	Median (Min-Max)
**Pregnant women**		
Age (years)		﻿31.5 (19–43)
<20 20–35 > 35	1 (4%﻿) 13 (52%﻿) 11 (44%)﻿	
Parity		
Primiparous Multiparous	8 (32%) 17 (68%)	
Occupation		
Seller Teacher Housewife Private employee Government employee	1 (4%) 1 (4%) 15 (60%) 6 (24%) 2 (8%)	
Education		
Elementary School Junior High School Senior High School University	3 (12%) 6 (24%) 10 (40%) 6 (24%)	
**Healthcare providers**		
Gender		
Male Female	1 (16.7%) 5 (83.3%)	
Age (years)		﻿41 (34–52)
20–35 > 35	1 (16.7%) 5 (83.3%)	
Working experience		
5–10 years >11 years	2 (33.3%) 4 (66.7%)	
Education		
Bachelor of Public Health Medical Doctor Diploma of Midwifery Bachelor of Nursing	1 (16.7%) 1 (16.7%) 3 (50%) 1 (16.7%)	
Occupation		
Head of PHC Medical Doctor Midwives Nurse	1 (16.7%) 1 (16.7%) 3 (50%) 1 (16.7%)	

**Table 2:** Participants demographic data

**Participant**	**Age**	**Parity**	**Education level**	**Occupation**
P1	36	Multiparous	Senior High School	Housewife
P2	29	Multiparous	Bachelor’s degree	Private employee
P3	36	Primiparous	Senior High School	Housewife
P4	31	Multiparous	Bachelor’s degree	Government employee
P5	41	Multiparous	Elementary School	Housewife
P6	28	Primiparous	Senior High School	Housewife
P7	27	Primiparous	Bachelor’s degree	Government employee
P8	39	Multiparous	Senior High School	Private employee
P9	24	Primiparous	Senior High School	Housewife
P10	32	Multiparous	Elementary School	Housewife
P11	22	Primiparous	Bachelor’s degree	Private employee
P12	40	Multiparous	Junior High School	Seller
P13	19	Primiparous	Senior High School	Housewife
P14	30	Multiparous	Junior High School	Housewife
P15	36	Multiparous	Bachelor’s degree	Private employee
P16	23	Primiparous	Senior High School	Private employee
P17	39	Multiparous	Junior High School	Housewife
P18	34	Multiparous	Junior High School	Housewife
P19	37	Multiparous	Junior High School	Housewife
P20	40	Multiparous	Senior High School	Private employee
P21	27	Primiparous	Senior High School	Housewife
P22	38	Multiparous	Bachelor’s degree	Teacher
P23	29	Multiparous	Junior High School	Housewife
P24	31	Multiparous	Senior High School	Housewife
P25	43	Multiparous	Elementary School	Housewife
**Participant**	**Age**	**Gender**	**Education**	**Occupation**
P26	34	Female	Bachelor of Nursing	Nurse
P27	52	Male	Bachelor of Public Health Science	Head of PHC
P28	45	Female	Medical Doctor	Doctor
P29	38	Female	Diploma of Midwifery	Midwife
P30	39	Female	Diploma of Midwifery	Midwife
P31	43	Female	Diploma of Midwifery	Midwife

### Themes

The themes emerged out of findings from in-depth interviews, focus group discussions, and field notes which were acquired from observations at the Primary Health Centers. Using the Three Delays Model by Thaddeus and Maine as the study framework, the researchers identified.

three themes: (1) Fear of being infected with COVID-19, (2) The stay-at-home policy, and (3) Re-designed ANC services. Each theme was broken into sub-themes and the descriptive quotes for each sub-theme are illustrated in Table 3.

**Table 3:** Summary of themes and sub-themes from the transcribed data

Themes	Sub-themes
1. Fear of being infected with COVID-19	Anxiety
	Perceived vulnerability
	The desire to protect self and loved ones
2. The stay-at-home policy	Transport barriers to healthcare
	Social activity restriction
3. Re-designed ANC services	ANC adjustments
	Focus on high-risk pregnancy
	Insufficient information
	COVID-19 preventive behaviors adherence

### 1. Fear of being infected with COVID-19

According to Three Delays Models, the first delay is related to the decision to seek care. During the pandemic time, pregnant women often prefer to delay their ANC visit because they are afraid of being infected with COVID-19. This theme is comprised of three sub-themes: anxiety, perceived vulnerability, and the desire to protect self and loved ones.

#### Anxiety

Almost all of participants stated that the pandemic had made them feel anxious. Interacting with people outside the home induced anxiety of being infected with COVID-19.

…I am very worried since I am pregnant … my office colleague was confirmed as having COVID-19 so I feel very anxious to have contact with other people including healthcare providers … (P8, multiparous, 39 years old).

Many participants worried about their health condition because of their weak immunity status and knew that their risk of contracting the virus increased when they visited healthcare facilities for ANC.

… I am scared because the immunity status of pregnant women is decreased and the COVID-19 virus spread very quickly, I really feel fear when I go to the PHC to attend ANC so, I prefer to delay my next ANC schedule … (P23, multiparous, 29 years old).

#### Perceived vulnerability

Another participant expressed having mixed feelings about getting pregnant at this time. She felt grateful because she had been trying to get pregnant for a long time. However, she knew that the fact she was pregnant during the COVID-19 pandemic made her more vulnerable and this hindered her ANC attendance.

… I’m grateful and happy for my first pregnancy after having waited for a long time … however I’m a vulnerable person because I am pregnant during the COVID-19 pandemic, so I do not attend ANC regularly … I hope to contact healthcare providers using social media … (P3, primiparous, 36 years old).

#### The desire to protect self and loved ones

Some participants lived with members of other vulnerable groups such as the elderly, children, and people with co-morbidities. Some participants feared that engaging in activities outside the home increased their chances of being infected and they tried to minimize visiting places they deemed high-risk, such as clinics where ANC was carried out. Some participants preferred to delay ANC to prevent themselves or their children and parents from getting ill.

… I have to minimize visiting high-risk transmission places because I have children and my parents have co-morbidities. We have to take care of each other … I will delay my ANC until the COVID-19 situation improves … (P8, multiparous, 39 years old).

### 2. The stay-at-home policy

According to Three Delays Models, the second delay is related to identify and reach the health facility. During the COVID-19 pandemic, pregnant women prefer to delay their ANC visit due to Large Social Scale Restriction policy by Indonesia Government. This theme is comprised of two sub-themes: transport barriers to health care and restriction of social activities.

#### Transport barriers to health care

Participants disclosed that the COVID-19 pandemic had created several barriers to their daily activities. The Indonesian government’s policy encouraged people to stay at home during the pandemic. Participants therefore faced public transport barriers to attending their ANC.

… I faced difficulties attending ANC during the COVID-19 pandemic because of a lack of public transport due to the government’s stay at home policy … it would be really helpful if I could have my healthcare consultations over social media … (P14, multiparous, 30 years old).

#### Social activity restriction

Almost all participants described experiencing social activity restrictions during the pandemic due to government policies. Participants were encouraged to stay at home and not to attend activities involving a lot of people to prevent transmission.

… My husband did not allow me to join social gatherings, recitations, or religious meetings during the pandemic … I had to follow the government’s regulations and stay at home, and so I decided not to attend ANC this month … (P6, multiparous, 28 years old).

### 3. Re-designing ANC services

According to Three Delays Models, the third delay is related to receive appropriate treatment at the health facility. Health care providers re-design ANC services during the COVID-19 pandemic. This theme is comprised of four sub-themes: ANC adjustments; focus on high-risk pregnancy; insufficient information; and adherence to COVID-19 preventive behaviors.

#### ANC adjustments

All participants experienced some ANC services being modified during the pandemic. Many services were re-designed to reduce COVID-19 transmission. This meant ANC sessions were shortened and pregnancy classes were cancelled.

… They asked me some questions such as whether I had flu-like symptoms or fever, then they measured my blood pressure and weight and assessed the fetus in my womb very quickly … the midwife did a quick physical examination … (P9, primiparous, 24 years old).

Healthcare providers confirmed that ANC procedures were adjusted to prevent transmission. The Indonesia Ministry of Health ANC adaptation guidance called for early screening, the separation of healthy women suspected of being pregnant, and modified seating and room arrangement.

… We provide ANC using a new adaptation era guidance from the Ministry of Health. We take pregnant women’s’ temperatures, assess symptoms such as coughs and colds, and ask their history before carrying out the physical examination … we adjusted the ANC procedures and duration to prevent COVID-19 transmission … we also canceled prenatal classes during the pandemic … (P30, midwife, 39 years old).

#### Focus on high-risk pregnancy

Healthcare providers said that the ANC examinations during the pandemic focused on patients’ health problems and complaints, meaning that pregnant women with no problems were more likely to delay ANC to minimize risk. Healthcare providers made home visits to women with high-risk pregnancies to keep them safe.

… We re-arranged ANC schedules for low-risk pregnancies to prevent infection and we made home visits to high-risk pregnant women to keep them at home … (P31, midwife, 43 years old).

… The midwife came to my house and did a physical examination. She explained that I should stay at home during this time and told me she would visit again in the following months to keep me safe … (P20, multiparous, 40 years old).

#### Insufficient information

All pregnant participants said they had received insufficient information during their ANC appointments. They maintained that ANC assessments were short in duration and that there was no discussion or health promotion given by healthcare providers. They were given no chance to express their feeling or ask questions related to their pregnancies, and they said they used social media to facilitate discussions with their healthcare providers and get pregnancy-related advice.

… when I had my ultrasound examination, the doctor did it quickly and did not explain the results in detail … he just said it was good and normal. The doctor didn’t give me the opportunity to express my feelings or ask questions about my pregnancy … (P10, multiparous, 32 years old).

… I met the midwife in the ANC room for a very short time … she did a quick physical examination, did not give much information about the examination results, and did not explain about my fetus in detail … maybe I can use social media to communicate with healthcare providers and get some pregnancy-related advice … (P11, primiparous, 22 years old).

#### Adherence to COVID-19 preventive behaviors

All participants said that they adhered to COVID-19 preventive behaviors when going outside the home. They wore masks, washed their hands, and observed physical distancing when attending ANC. There is a big banner containing information about COVID-19 preventive measures in front of the PHC office.

… I always wear a mask, wash my hands in the wash basin which placed in front of the PHC, and sit down not too close to other patients in the PHC … there is a person in charge who always reminds us to wear a mask, wash our hands, and keep a safe distance from other people … (P14, multiparous, 30 years old).

Healthcare providers confirmed that there is a banner in front of the PHC that informs patients about COVID-19 preventive behaviors. They maintained that they frequently reminded patients to follow the preventative measures, provided a hand washing basin outside the PHC, and wore additional personal protective equipment.

…. We provide healthcare services in the PHC using a high standard of COVID-19 preventive measures … There is a big banner in front of the PCH informing patients of the COVID-19 preventive behaviors … we always remind patients to wear a mask, keep at least two meters distance from others, and wash their hands using water and soap before and after entering the PHC … healthcare providers should wear additional personal protective equipment … (P2, head of PHC, 52 years old).

## Discussion

To our knowledge, this is the first Indonesian study to investigate the barriers to ANC from the perspectives of pregnant mothers and healthcare providers during the COVID-19 pandemic. Despite facing many challenges and restrictions throughout the pandemic, healthcare providers have had to continue providing a full range of ANC services to the pregnant population. Some services were modified in order to prevent COVID-19 transmission [33]. The number of ANC visits decreased during the pandemic, particularly among pregnant women in rural areas [33]. This might have been due to fear of infection, transport problems, the fact that women lived far away from health facilities, the government’s COVID-19 policies, changing healthcare services, or lack of awareness about the benefits of ANC.

Our findings show that many pregnant women were afraid of COVID-19 infection. Pregnant woman are particularly vulnerable to viruses due to changes to maternal immune responses and increased sensitivity to respiratory pathogens [18]. Most pregnant women in this study attended ANC in PHCs however, this made them feel anxious. Our results are similar to those of a previous study that found that pregnant women during the pandemic missed ANC visits and were afraid of giving birth in health facilities due to a fear of contracting COVID-19 [34]. Our results also correlate to those of another study that discovered that while women desired to self-monitor their pregnancies, they did not have sufficient knowledge, and that they felt the need to strictly isolate at home and expected pregnancy tele-consultations [35]. In our study, fear was the most important factor in reduced ANC during the pandemic, and this correlates to the findings of a study carried out in Israel, whereby women refused to visit healthcare centers for ANC out of fear of viral infection [36]. A large survey found that pregnant women were particularly fearful of restrictions to family visits post-delivery, the possibility of their babies being infected COVID-19, the lack of support offered during delivery, and delivery plans changing [37]. Anxiety related to COVID-19 transmission was also experienced by healthcare workers in Finland, particularly among women and young people [38]. Unsurprisingly, our results show that the combination of pregnancy and working status increased the fear of viral transmission among Indonesian working women who conceived during the pandemic.

Our study shows that Indonesian pregnant women were not only scared of COVID-19 infecting themselves but also worried that they would transmit the virus to family members with comorbidities since most participants lived with their extended families. Pregnant women in Iran also experienced fear, obsession, boredom, nervousness, and despair during the pandemic due to the highly contagious nature of the virus [39]. Living with the extended family is common for people living in rural areas of Indonesia. The majority of participants in this study lived with their parents, parents-in-law, or grandparents, many of whom had comorbidities. Fear of COVID-19 transmission among people living in rural areas may have been caused by low adherence to healthcare protocols. Participants in this study said they felt uncomfortable wearing masks and wanted to join their neighbors for social interactions. Urban dwellers in other studies found it easier to share knowledge about COVID-19 [40], advised others to carry out preventive behaviors [40], and were found to have less negative attitudes regarding preventive behaviors [41]. The low adherence of COVID-19 preventive behaviors among people in rural areas might be due to low health literacy and lack of information. One study found that people with adequate knowledge of the virus were more likely to engage in appropriate preventive behaviors and that urban dwellers were more likely to practice good preventive behaviors than rural dwellers [42].

The qualitative findings of this study further illustrate that the pandemic affected public transport and restricted social activities. The Indonesian government restricted non-essential travel and group activities to prevent the spread of COVID-19 [43]. Participants in this study reported that public transport was limited due to government policies and so they found it difficult to attend their ANC appointments. These findings are similar to those reported in a previous study that also found that pregnant women faced difficulty accessing public transport pandemic, resulting in missed ANC appointments [44]. Social support from family members, friends, and significant others contributes to developing resilience among pregnant women, positively affecting perinatal mental health and obstetric outcomes [45]. Social activities are part of daily life for people living in Indonesia’s rural areas. People are accustomed to visiting neighbors, praying together in the mosque, and attending funerals, recitations, wedding ceremonies, and social gatherings.

The Ministry of Health launched perinatal and newborn health service guidelines to guide healthcare providers in the delivery of healthcare services during the pandemic. The guidelines included information regarding general precautions, health facility readiness, perinatal care recommendations, and health services for maternal and newborns [33]. Healthcare providers in this study described having to redesign ANC programs, limit prenatal examinations, carry out more home visits, and limit the number of prenatal class participants. These ANC program adjustments were made to protect both healthcare providers and pregnant women from COVID-19 transmission, particularly during July 2021 when daily cases in Indonesia reached the highest level [3]. Unsurprisingly, the situation of maternal health services during a pandemic is more precarious in developing countries due to insufficient infrastructure and resources, the collapse of healthcare systems, decreased workforce, access decline, and fewer ANC visits [46]. Consistent with a study in Kenya that reported ANC program modifications including limiting ANC hospital appointments and increasing networking between community health workers and communities [47]. Participants in this study reported that they feel unsatisfied because they did not have sufficient time to discuss their pregnancies with healthcare providers during their ANC appointments. Participants in another study reported that if they stated dissatisfaction with ANC services due to changes caused by the pandemic, they had their ANC appointments and pregnancy classes cancelled or suspended [48]. One study found that pregnant women were not examined in detail during ANC appointments, and that this may have led to health professionals missing important aspects in their pregnancy [44]. Participants in the same study also used social media or written documents to obtain information from their healthcare providers during the pandemic. Pregnant women in another study declared social media a useful tool in providing antenatal care and support during the pandemic, particularly with regard to obtaining pregnancy-related information, managing feelings of isolation, service specific issues, and routine care [49]. The use of social media is an interesting way to communicate and deliver health promotion to pregnant women, as identified in this study.

In another study, good preventive behavior among pregnant women was found to be significantly associated with fear of transmission and good knowledge of COVID-19 preventive behaviors [50]. Participants in this study understand that pregnant women are a vulnerable group due to their lowered immune response, and they rigorously implemented preventive measures such as wearing masks, washing hands with water and soap, and observing physical distancing, even while attending ANC appointments in the PHC. Such careful adherence to COVID-19 preventive behaviors may be due to the government’s massive publicity campaign.

## Strengths and limitations

A strength of this study is that it used open-ended questions which provided rich data for qualitative analysis. Its findings are still relevant to improving maternal health services. Despite its strengths, this study also has limitations. Its principal limitation is that the findings are of limited generalizability since all participants were married women of Javanese ethnicity who attended ANC appointments during the pandemic. Further research is required to examine the utility of social media as way of communicating between healthcare providers and pregnant women.

## Conclusion

This study highlights the substantial impact COVID-19 had on ANC in rural areas of Indonesia. Its findings contribute to the growing body of evidence outlining the challenges faced in providing ANC in rural areas during pandemics. Pregnant women faced several difficulties in seeking antenatal care, reaching antenatal care facilities, and receiving satisfactory services at the health care facilities during the COVID-19 pandemic.

## Data Availability

The datasets generated during and/or analyzed during the study are available from the corresponding author on reasonable request.

## References

[1] World Health O. COVID 19 Public Health Emergency of International Concern (PHEIC). Global research and innovation forum: towards a research roadmap. 2020.

[2] WHO. WHO coronavirus disease (COVID-19) dashboard with vaccination data: Indonesia 2021 [Available from: https://covid19.who.int/region/searo/country/id.

[3] Worldmeter. Worldmeter’s COVID-19 data: Indonesia 2021 [Available from: https://worldmeters.info/coronavirus/country/indonesia/.

[4] Read S, Wild SH. What are the risks of COVID-19 infection in pregnant women? 2020; 395:[760-2 pp.]. 10.1016/S0140-6736(20)30365-2 PMC7158939 32151334

[5] Health IMo. Indonesia Health Profile. In: Health Mo, editor. Jakarta2018.

[6] Health IMo. Indonesia Health Profile. In: Health Mo, editor. Jakarta2019.

[7] Health IMo. Indonesia Health Profile. In: Health Mo, editor. Jakarta2020.

[8] Nasional BKdKB. Kematian ibu hamil selama pandemi tinggi, Kepala BKKBN apresiasi kecepatan vaksinasi di DKI Jakarta2021. Available from: https://www.bkkbn.go.id/detailpost/kematian-ibu-hamil-selama-pandemi-tinggi-kepala-bkkbn-apresiasi-kecepatan-vaksinasi-di-dki-jakarta.

[9] Carvalho-Sauer R, Costa M, Teixeira M, Nascimento E, Silva E, Barbosa M et al. Impact of COVID-19 pandemic on time series of maternal mortality ration in Bahia, Brazil: analysis of period 2011–2020.BMC pregnancy and childbirth. 2021;21. 10.1186/s12884-021-03899-y PMC8190975 34112099

[10] Tola W, Negash E, Sileshi T, Wakgari N (2021). Late initiation of antenatal care aand associated factors among pregnant women attending antenatal clinic of Ilu Ababor Zone, Southwest Ethiopia: a cross-sectional study. PLoS ONE.

[11] UNICEF. Antenatal Care. In: UNICEF, editor. 2021.

[12] Abbas A, Rabeea M, Hafiz H, Ahmed N, editors., editors. Effects of irregural antenatal care attendace in primiparas on the perinatal outcomes: a cross sectional study. Proceedings in Obstetrics and Gynecology; 2017.

[13] Mckinney E (2018). Maternal-child nursing.

[14] Kazemi SN, Hajikhani B, Didar H, Hosseini SS, Haddadi S, Khalili F et al. COVID-19 and cause of pregnancy loss during the pandemic: A systematic review.Plos One. 2021:1–10. 10.1371/journal.pone.0255994 PMC8357105 34379700

[15] Westgren M, Pettersson K, Hagberg H, Ganesh A (2020). Severe maternal morbidity and mortality associated with COVID-19: the risk should not be downplayed. Acta Obstet Gynecol Scand.

[16] Goyal M, Singh P, Singh K, Shashank S, Agrawal N, Misra S. The effect of COVID-19 pandemic on maternal health due to delay in seeking health care: experience from a tertiary center. International Journal of Gynecology & Obstetrics; 2020. 10.1002/ijgo.13457 PMC9087665 33128794

[17] Organization) PPAH. Epidemiological alert: COVID-19 during pregnancy Washington D.C2020 [Available from: https://www.paho.org/en/news/4-8-2020-paho-director-warns-disruptions-regular-health-services-due-covid-19.

[18] Liu H, Wang L-L, Zhao S-J, Kim JK, Mor G, Liao A-H (2020). Why are pregnant women susceptible to COVID-19? An immunological viewpoint. J Reprod Immunol.

[19] Zacharias P, Joseph M, Jacob A, Viji CLM, Josy N, Lydia S (2021). Barriers to antenatal care during COVID-19 pandemic: a hospital-based retrospective cross-sectional study in rural South Karnataka. The New Indian Journal of Obgyn.

[20] Hailemariam S, Agegnehu W, Misganaw D, Exploring (2021). COVID-19 related factors of influencing antenatal care services uptake: a qualitative study among women in rural community in Southwest Ethiopia. J Prim Care Community Health.

[21] Singh DR, Sunuwar DR, Shah SK, Karki K, Sah LK, Adhikari B (2021). Impact of COVID-19 on health services utilization in Province-2 of Nepal: a qualitative study among community members and stakeholders. BMC Health Serv Res.

[22] Meaney S, Leitao S, Olander EK, Pope J, Matvienko-Sikar K (2022). The impact of COVID-19 on pregnant womens’ experiences and perceptions of antenatal maternity care, social support, and stress-reduction strategies. Women Birth.

[23] McDonald CR, Weckman AM, Wright JK, Conroy AL, Kain KC (2020). Pregnant women in low-and middle-income countries require a special focus during the COVID-19 pandemic. Front Global Women’s Health.

[24] Polit DF, Beck CT (2018). Essentials of nursing research: appraising evidence for nursing practice.

[25] Kim H, Sefcik JS, Bradway C (2017). Characteristics of qualitative descriptive studies: a systematic review. Res Nurs Health.

[26] Thaddeus S, Maine D. Too far to walk: maternal mortality in context. Social science & medicine (1982). 1994;38(8):1091 – 110. 10.1016/0277-9536(94)90226-7 8042057

[27] Wulandari RD, Laksono AD, Rohmah N (2021). Urban-rural disparities of antenatal care in South East Asia: a case study in the Philippines and Indonesia. BMC Public Health.

[28] Statistik BP. Profil Penduduk Indonesia Hasil SUPAS. Badan Pusat Statistik Jakarta. 2015.

[29] Pitoyo AJ, Triwahyudi H (2017). Dinamika perkembangan etnis di Indonesia dalam konteks persatuan negara. Populasi.

[30] Suminar YD, Lutiarsi RT, Wibowo MA, Lestari ES (2021). Profil Kesehatan Jawa Tengah.

[31] Braun V, Clarke V (2006). Using thematic analysis in psychology. Qualitative Res Psychol.

[32] Tong A, Sainsbury P, Craig J (2007). Consolidated Criteria for reporting qualitative research (COREQ): a 32-Item Checklist for interviews and focus groups. Int J Qual Health Care.

[33] Health IMo (2020). Guidelines for Antenatal Care, Labor, Postpartum, and Newborn in New Normal Era.

[34] Moyer CA, Sakyi KS, Sacks E, Compton SD, Lori JR, Williams JEO (2020). COVID-19 is increasing ghanaian pregnant women’s anxiety and reducing Healthcare seeking. Int J Gynecol Obstet.

[35] Ge Y, Shi C, Wu B. Anxiety and Adaptation of Behavior in Pregnant Zhuang Women During the COVID-19 Pandemic: A Mixed-Mode Survey.Risk Management and Healthcare Policy. 2021;14. 10.2147/RMHP.S303835 PMC8055250 33883960

[36] WHO, Coronavirus. and pregnancy preserving maternal health across the european region 2020 [Available from: https://www.euro.who.int/en/health-topics/Life-stages/maternal-and-newborn-health/news/news/2020/6/coronavirus-and-pregnancy-preserving-maternal-health-across-the-european-region.)

[37] Basu A, Kim HH, Basaldua R, Choi KW, Charron L, Kelsall N et al. A Cross-National Study of Factors Associated with Women’s Perinatal Mental Health and Wellbeing During the COVID-19 Pandemic.Plos Ones. 2021:1–18. 10.1371/journal.pone.0249780 PMC8059819 33882096

[38] Savolainen L, Oksa R, Savela N, Celuch M, Oksanen A (2021). COVID-19 Anxiety- A longitudinal survey study of psychological ad situational risks among finnish worker. Int J Environ Res Public Health.

[39] Bakouei F, Nikpour M, Rad HA, Marzoni ZA. Exploration of the Pregnant Women’s Experiences During COVID-19 Disease Crisis: A Qualitative study. 2020.

[40] Zhang J, Zhu L, Li S, Huang J, Ye Z, Wei Q (2021). Rural-urban DIsparities in knowledge, Behaviors, and Mental Health during COVID-19 pandemic. Medicine.

[41] Chen X, Chen H (2020). Differences in preventive ehaviors of COVID-19 between urban and rural residents: lessons learned from a cross-sectional study in China. Int J Environ Res Public Health.

[42] Gutu B, Legese G, Fikadu N, Kumela B, Shuma F, Mosisa W (2021). Assessment of preventive behavior and associated factors towards COVID-19 in Qellam Wallaga Zone, Oromia, Ethiopia: a community-based cross-sectional study. PLoS ONE.

[43] Health IMo. Abolition of mudik at ied fitr 1442 and preventing corona virus disease 2019 (COVID-19) during ramadhan 1442. In: Health Mo, editor. Jakarta2021.

[44] Karavadra B, Stockl A, Prosser-Snelling E, Simpson P, Morris E (2020). Women’s perceptions of COVID-19 and their healthcare experiences: a qualitative thematic analysis of a national survey of pregnant women in the United Kingdom. BMC Pregnancy Childbirth.

[45] Dunkel C (2011). Psychological science on pregnancy: stress process, biopsychosocial models, and emerging research issues. Ann Rev Psychol.

[46] Tadesse E (2020). Antenatal Care Service utilization of pregnant women attending Antenatal Care in Public Hospitals during the COVID-19 pandemic period. Int J Womens Health.

[47] Kimani RW, Maina R, Shumba C, Shaibu S (2020). Maternal and newborn care during the COVID-19 pandemic in Kenya: recontextualising the community midwifery model. Hum Resour Health.

[48] Meaney S, Leitao S, Pope J, Matvienko-Sikar K. The impact of COVID-19 on pregnant women’s experiences and perceptions of antenatal maternity care, social support, and stress-reduction strategies.Women Birth. 2021(21). 10.1016/j.wombi.2021.04.013 PMC9051126 33994134

[49] Chatwin J, Butler D, Jones J, James L, Choucri L, McCarthy R (2021). Experiences of pregnant mothers using a social media based antenatal support service during the COVID-19 lockdown in the UK: findings from a user survey. BMJ open.

[50] W M, Kassie GT, Asratie B, Abate M (2021). The effects of fear and knowledge of COVID-19 on preventive practice among pregnant women who attend antenatal care in Northwest Ethiopia, 2020: institution-based cross-sectional study. Int J Women’s Health.

